# Mental Disease Following Influenza

**Published:** 1909-09-11

**Authors:** 


### Mental Diseases.

### MENTAL DISEASE FOLLOWING INFLUENZA.

The pathological relationship between insanity and the influenza bacillus has not yet been determined, though clinically some cases of mental breakdown seem to be due to the toxic effects of the microbic infection. In a great many cases with a history of the complaint it is the only discoverable ætiological factor; whilst in others it appears to be either the determining or the predisposing cause. Where it is the determining cause, the history given is that for some time the patient had a good deal of unusual mental stress—domestic, financial, or business worries—but that there was no indication of mental abnormality until an attack of influenza. The physical disability accompanying the attack, however, seemed to be the last straw, and he appeared suddenly to give up fighting against odds and become despondent. Where it is the predisposing cause, the friends report that after the attack the patient found that he was unable to do his ordinary work as well as before, or without much effort; and that this seemed to cause him a good deal of distress, which led to the first mental symptoms. The first signs of definite insanity may be many months after the influenza, but it is usually reported that the patient has never been himself since the attack, though it is often very difficult to get a description of exactly how the patient has changed.

Another interesting point in connection with these cases is that the attack of influenza is usually a mild one, and uncomplicated by any other trouble. It is found, too, that the patient did not treat the illness seriously, not commencing any treatment until late, and going back to ordinary occupation too soon—two errors almost invariably committed by patients suffering from the milder forms of influenza. The reason that these errors are so commonly committed seems to be that the toxins formed in this disease cause more prostration, and subsequent weakness, of the higher nervous centres than of the lower ones; and that lessened ability to stand mental stress is not so readily recognised as lessened ability to stand physical stress. Further, even if patients do recognise that their ordinary occupation is causing them more mental strain than usual, they will generally overlook the danger, and refuse to take any precautions to relieve it. In diseases, however, in which the lower nervous centres are more affected than the higher, and the subsequent weakness during convalescence is physical rather than mental, the patient not only realises this weakness, but will take precautions for recovering from it.

Cases of mental disease following influenza resemble one another in many respects, though the type varies with associated ætiological factors. The patients are usually depressed, and show marked signs of general nervous debility. The earliest symptom, and one which is usually very persistent throughout, is insomnia. This is accompanied by much loss of tone, and by muscular tremors, constipation, dirty tongue and foul breath. Food is taken badly, and often the first indication of marked mental trouble is the appearance of delusions of suspicion against friends, due to an idea that food is being poisoned. Loss of self-control is noticed early in the attack, and often determined attempts at suicide are made. Hallucinations of any of the senses may appear, and delusions of all kinds. Sometimes the symptoms are of the maniacal type, and these also have physical symptoms of debility.

Recovery takes place in most cases, though the prognosis is not so good in patients with a family history of insanity. As regards the duration of the attack, it is unsafe to prophesy; but it is always well to insist on a prolonged period of convalescence, during which all anxiety and worry must be strictly avoided. Breakdowns are frequent in cases that are allowed to return to work too soon, and the relapses are often more serious than the original attack, besides disheartening both patient and friends. Some cases, instead of recovering, drift into a state of dementia, or become chronically deluded.

A great many of these cases could undoubtedly be caused to abort if proper care were taken by the patient. From the point of view, however, of prophylaxis, the need of proper rest during and after mild influenza attacks cannot be too strongly insisted upon.

As soon as any signs of mental symptoms appear, or it is found that the patient is feeling pressure in his ordinary work, complete removal from all domestic and business anxieties must be insisted upon. This will nearly always necessitate leaving home—a step which will certainly be strongly opposed by the patient himself, and frequently by others as well. No compromise, however, should be countenanced if a quick recovery is desired. The first step in treatment should be taken at the same time—namely, to see that the intestinal canal is thoroughly cleared of any accumulations. To ensure this, 5 grs. of pil. hydrarg. should be given at night, followed by 2 drachms of phosphate of soda in the morning; and this should be repeated every day for four or five days, or even longer if necessary. This active treatment will usually effect a marked improvement, and the insomnia, if present, will begin to abate. If insomnia is a serious symptom and is disheartening the patient, hypnotics may be prescribed. The best of these is Paraldehyde, if it can be borne by the stomach; but, if not, then Veronal. Five grains of this latter will usually be sufficient, or 2 drachms of Paraldehyde. Hypnotics should be discontinued as soon as possible, and the patient should never be informed what drug is being given. In cases where the muscular tone is poor, massage will be found to be of value. Of tonics in the low nervous cases, Lecithin, if given for some time, is amongst the best; but it does not show any effect for some time as a rule. Great care must be taken to prevent the patient going back to work until he is thoroughly well and has had a sufficient rest: neglect of this point is the most frequent cause of relapses.

